# Role of the Ste20‐like kinase SLK in podocyte adhesion

**DOI:** 10.14814/phy2.15897

**Published:** 2024-01-01

**Authors:** Andrey V. Cybulsky, Joan Papillon, Craig Bryan, José R. Navarro‐Betancourt, Luc A. Sabourin

**Affiliations:** ^1^ Department of Medicine McGill University Health Centre Research Institute, McGill University Montreal Quebec Canada; ^2^ Ottawa Hospital Research Institute, Cancer Therapeutics Ottawa Ontario Canada

**Keywords:** focal adhesion kinase, glomerulonephritis, paxillin, Talin‐1, vinculin

## Abstract

SLK controls the cytoskeleton, cell adhesion, and migration. Podocyte‐specific deletion of SLK in mice leads to podocyte injury as mice age and exacerbates injury in experimental focal segment glomerulosclerosis (FSGS; adriamycin nephrosis). We hypothesized that adhesion proteins may be substrates of SLK. In adriamycin nephrosis, podocyte ultrastructural injury was exaggerated by SLK deletion. Analysis of a protein kinase phosphorylation site dataset showed that podocyte adhesion proteins—paxillin, vinculin, and talin‐1 may be potential SLK substrates. In cultured podocytes, deletion of SLK increased adhesion to collagen. Analysis of paxillin, vinculin, and talin‐1 showed that SLK deletion reduced focal adhesion complexes (FACs) containing these proteins mainly in adriamycin‐induced injury; there was no change in FAC turnover (focal adhesion kinase Y397 phosphorylation). In podocytes, paxillin S250 showed basal phosphorylation that was slightly enhanced by SLK; however, SLK did not phosphorylate talin‐1. In adriamycin nephrosis, SLK deletion did not alter glomerular expression/localization of talin‐1 and vinculin, but increased focal adhesion kinase phosphorylation modestly. Therefore, SLK decreases podocyte adhesion, but FAC proteins in podocytes are not major substrates of SLK in health and disease.

## 1 INTRODUCTION

Mechanisms underlying hereditary and acquired glomerulopathies converge on disruption of the cytoskeleton in podocytes/glomerular epithelial cells (GECs). Regulatory networks of signaling factors tightly control the podocyte actin cytoskeleton, and even minor changes can shift the system toward podocyte disease (Schell & Huber, 2017; Tian & Ishibe, 2016; Welsh & Saleem, 2012). Podocytes are highly differentiated cells, critical for the maintenance of glomerular permselectivity (Greka & Mundel, 2012; Pavenstadt et al., 2003). They have a complex morphology featuring interdigitating foot processes that are bridged by filtration slit diaphragms, and they adhere to the glomerular basement membrane (GBM). In the apical domain of the foot process, podocalyxin and other proteins maintain negative surface charges that repel proteins and act as spacers preventing foot process effacement (Doyonnas et al., 2001; Neal, 2015; Wharram et al., 2000). Podocalyxin global knockout (KO) mice fail to form foot processes and die after birth (Doyonnas et al., 2001). Podocyte‐specific podocalyxin KO mice develop focal segmental glomerulosclerosis (FSGS) and nephrotic syndrome (Refaeli et al., 2020). The cytoskeletal protein ezrin, phosphorylated at T567 (an activation‐specific phosphorylation site), is believed to be important for podocalyxin function, as it links F‐actin to the C‐terminus of podocalyxin directly or via Na/H exchanger regulatory factor 2 (NHERF2) (Al‐Momany et al., 2014; Takeda et al., 2001). In the basal/slit diaphragm region of the podocyte (Neal, 2015), cortical actin is connected to nephrin and other slit diaphragm components, as well as to integrins through proteins that are components of focal adhesion complexes (FACs) (Lennon et al., 2014; Pavenstadt et al., 2003; Schell & Huber, 2017; Tian & Ishibe, 2016; Welsh & Saleem, 2012).

Podocyte adhesion to the GBM is, in part, mediated by β1‐integrins, a heterodimeric family of transmembrane adhesion molecules (Lennon et al., 2014; Sachs & Sonnenberg, 2013; Schell & Huber, 2017; Tian & Ishibe, 2016). Based on studies mainly in cultured cells, integrins connect with the actin cytoskeleton via FACs—dynamic anchoring points for cell attachment and communication with the actin cytoskeleton. Among FAC proteins, talin‐1 (subsequently “talin”) contains binding sites for β1‐integrin, F‐actin, vinculin, and focal adhesion kinase (FAK). Talin amplifies the affinity of the integrin for adhesion, and recruits proteins, including paxillin, to FACs (Goult et al., 2018; Lennon et al., 2014; Schell & Huber, 2017; Tian et al., 2014). Vinculin interacts with α‐actinin, actin, paxillin and other proteins, and it modulates integrin clustering and integrin‐actin interaction (Lausecker et al., 2018; Lennon et al., 2014). Paxillin is an important molecular adaptor and a scaffolding protein for vinculin, talin, and other proteins within FACs (Lennon et al., 2014). These FAC proteins are believed to be regulated by phosphorylation. FAK is a nonreceptor tyrosine kinase and integrin‐induced, activation‐specific autophosphorylation of FAK at Y397 stimulates signaling pathways required for FAC assembly and turnover. Thus, FAC turnover has been correlated with decreased levels of phospho‐FAK Y397, whereas stable FACs show persistently elevated levels (Garland et al., 2021). In mice, podocyte‐specific KO of β1 or α3 integrin, or talin leads to proteinuria, foot process effacement, and early death (Kreidberg et al., 1996; Lennon et al., 2014; Pozzi et al., 2008; Tian et al., 2014). Mice with podocyte‐specific vinculin KO show a milder phenotype (Lausecker et al., 2018), while podocyte‐specific FAK KO mice are healthy and are resistant to foot process effacement in glomerulonephritis (Ma et al., 2010).

Hereditary and acquired glomerulopathies often feature podocyte injury (foot process effacement, retraction, detachment from the GBM), proteinuria, and eventual glomerulosclerosis and impaired glomerular function (Chiang & Inagi, 2010; Cybulsky, 2011). Foot process effacement is associated with broad, firm adhesion of podocytes to the GBM, and may represent a reactive, more primitive epithelial phenotype that enhances adhesion to the GBM and limits the risk of detachment, at least temporarily (Kriz et al., 2013). Thus, healthy podocytes contain noncontractile actin cables in the foot processes that are connected to contractile actin cables located in the major processes and the cell body. Podocyte injury leads to the disassembly of the noncontractile actin cables in the foot processes and appearance of sarcomere‐like contractile actin cables juxtaposed to the GBM (actin “mat”), and possibly enhanced adhesion (Schell & Huber, 2017; Suleiman et al., 2017). Detachment of podocytes from the GBM may be counteracted by spreading or migration of remaining podocytes to cover denuded GBM, but this view requires further validation. In humans, mutations in numerous podocyte cytoskeletal, slit diaphragm or apical membrane proteins, GBM collagens and Rho GTPase regulators can lead to podocyte injury, proteinuria and FSGS (Akchurin & Reidy, 2015; Chiang & Inagi, 2010; Greka & Mundel, 2012; Neal, 2015; Rosenberg & Kopp, 2017; Schell & Huber, 2017; Yao et al., 2019). Acquired human FSGS is likely initiated by a circulating factor that is toxic to podocytes (Rosenberg & Kopp, 2017). Among well‐established animal models of podocyte injury, mice that express an actinin‐4 K256E transgene in podocytes recapitulate human hereditary FSGS (Cybulsky & Kennedy, 2011), while adriamycin nephrosis is a prototypical experimental model of acquired human FSGS (Pippin et al., 2009).

The Ste20‐like kinase SLK is a serine/threonine kinase that contributes to the maintenance of healthy podocyte structure and pathogenesis of experimental FSGS (Woychyshyn et al., 2020). SLK displays a complex structure and regulation (Al‐Zahrani et al., 2013; Garland et al., 2021), and is expressed ubiquitously in adult tissues (Al‐Zahrani et al., 2013, 2014). Global deletion of SLK in mice results in severe developmental defects and death at embryonic day 14.5 (Al‐Zahrani et al., 2014; Pryce et al., 2017). In mice, SLK participates in myoblast differentiation, and muscle‐specific deletion results in myopathy (Pryce et al., 2017; Storbeck et al., 2013). SLK may control cell cycle progression, actin stress fiber assembly, cell adhesion, spreading, and migration in tumor cells, fibroblasts and GECs (Al‐Zahrani et al., 2013; Cybulsky et al., 2017; Garland et al., 2021). This diverse signaling of SLK may be regulated via distinct substrates, including protein kinases (Cybulsky et al., 2016; Hao et al., 2006) and cytoskeletal proteins, such ezrin (Cybulsky et al., 2017; Machicoane et al., 2014; Viswanatha et al., 2012) and paxillin (Quizi et al., 2013).

We produced mice with podocyte‐specific deletion of SLK (Cybulsky et al., 2018). Albuminuria developed at 4–5 months of age in males, and 8–9 months in females, and persisted for up to 13 months. At 11–12 months, synaptopodin, nephrin, and podocalyxin expression were reduced; there was podocyte depletion and glomerular ultrastructural damage, including focal foot process effacement and microvillous transformation of podocyte plasma membranes. More recently, we induced adriamycin nephrosis in 3–4‐month control and podocyte SLK KO mice (Woychyshyn et al., 2020). Compared to control, SLK deletion exacerbated albuminuria and loss of podocytes. We also showed that SLK can phosphorylate ezrin at T567 (Cybulsky et al., 2017), and deletion of SLK reduced the colocalization of ezrin and podocalyxin in the glomerulus (Woychyshyn et al., 2020). Thus, SLK deletion leads to podocyte depletion, injury and albuminuria, as mice age, and deletion exacerbates these parameters in adriamycin nephrosis. It is reasonable to conclude that SLK regulates podocyte integrity, at least in part, by maintaining apical structure, i.e., the podocalyxin‐ezrin‐F‐actin axis, which involves ezrin phosphorylation. However, in vivo knockdown of ezrin did not lead to podocyte injury or proteinuria (Hatano et al., 2018). Thus, we hypothesize that there may be other substrates of SLK in podocytes that potentially mediate glomerular injury. For example, SLK activation associated with FAK signaling and subsequent phosphorylation of paxillin at S250 by SLK was shown to be required for FAC turnover and efficient migration in fibroblasts. SLK was colocalized with paxillin at the leading edge of migrating fibroblasts (Quizi et al., 2013). In addition, the Drosophila SLK ortholog Slik phosphorylates talin at T152, and this is required for recruitment of talin to integrin adhesion sites, and maintenance of myofiber stability and muscle attachment (Katzemich et al., 2019). Given the reported association of SLK with FACs in migrating fibroblasts (Garland et al., 2021) and that the FAC components paxillin and talin were reported to be SLK substrates in fibroblasts (Quizi et al., 2013) and Drosophila muscle cells (Katzemich et al., 2019), respectively, we examined whether paxillin and talin may also be SLK substrates in podocytes, and if they are recruited to sustain podocyte structure. In the present study, we show that in cultured GECs, deletion of SLK resulted in changes in paxillin, vinculin, and talin FACs, in the context of adriamycin‐induced injury. There was modest paxillin S250 phosphorylation by SLK. However, there was no change in FAK Y397 phosphorylation, and we did not observe phosphorylation of talin by SLK. In adriamycin nephrosis, deletion of SLK resulted in a small increase in FAK Y397 phosphorylation.

## 2 MATERIALS AND METHODS

### 2.1 Materials

Mouse anti‐vinculin (clone V9131), rabbit anti‐paxillin (SAB4502553), rabbit anti‐integrin α3 (AB1920), and rabbit anti‐actin IgGs (A2066) were purchased from Millipore‐Sigma (Oakville, ON). Mouse anti‐human talin‐1 IgG (clone 97H6) was purchased from Bio‐Rad Laboratories (Mississauga, ON). Rabbit monoclonal antibody to phospho‐FAK Y397 (31H5L17) was purchased from ThermoFisher Scientific (Saint‐Laurent, QC). Rat anti‐collagen IV α5 clone H53 (7078) was purchased from Chondrex Inc. (Woodinville, WA). Rabbit anti‐nephrin antiserum was kindly provided by Dr. Tomoko Takano (McGill University). Fluorescein isothiocyanate‐conjugated AffiniPure goat anti‐rabbit IgG (#111‐095‐003), rhodamine‐conjugated AffiniPure goat anti‐mouse IgG (#415‐505‐1), peroxidase‐conjugated AffiniPure sheep‐anti mouse IgG (#515‐035‐003), and peroxidase‐conjugated AffiniPure goat anti‐rabbit IgG (#111‐035‐003) were purchased from Jackson Immunoresearch (West Grove, PA).

Electrophoresis reagents were from Bio‐Rad Laboratories (Mississauga, ON), and GE Healthcare (Baie d'Urfé, QC). Tissue culture media and Lipofectamine 2000 were from Wisent (Saint‐Jean‐Baptiste, QC) and Invitrogen‐Life Technologies (Burlington, ON).

### 2.2 Mouse studies

Production, breeding, and genotyping of podocyte‐specific SLK KO mice (SLK^flox/flox^; Cre/+) was described previously (Cybulsky et al., 2018). These mice carry a podocin‐Cre‐mediated deletion of exons 3–6 of *Slk* (Cre recombinase gene under the control of the podocin promoter). SLK^flox/flox^;+/+ mice constitute littermate controls. Mice were housed in a standard animal care facility with 12 h on–off light cycles, and were fed ad libitum. Adriamycin (doxorubicin) was administered intravenously via tail vein to the mice (Woychyshyn et al., 2020). After 4 weeks, mice were euthanized (isofluorane followed by cervical dislocation); kidneys were collected for microscopy, and glomeruli were isolated utilizing a differential sieving technique (Cybulsky et al., 2018). Animal protocols were approved by the McGill University Animal Care Committee. All methods were performed in accordance with the relevant guidelines and regulations.

For immunofluorescence (IF) microscopy, kidney poles were snap‐frozen in isopentane (−80°C). Cryostat sections (4 μm thickness) were cut and then fixed in 4% paraformaldehyde (22°C), ice‐cold methanol or ice‐cold acetone, and blocked with 5% normal rabbit or goat serum or 3%–5% BSA. Incubations with primary antibodies were performed overnight at 4°C, and incubations with secondary antibodies were 1 h at 22°C. In control incubations (performed in parallel), primary antibody was replaced with nonimmune IgG. In some experiments, cell nuclei were stained with Hoechst H33342. Images were acquired using a Zeiss Axio Observer Z1 LSM780 laser scanning confocal microscope with ZEN2010 software (McGill University Health Centre Research Institute Imaging Platform). To compare fluorescence intensities, all images were taken at the same exposure time. Fluorescence intensity was quantified using the histogram function of ImageJ software (National Institutes of Health, Bethesda, MD), and results are expressed in arbitrary units (Cybulsky et al., 2018). The glomerular fluorescence intensity was normalized to the total fluorescence in each image. To measure the colocalization of two proteins in mouse kidney sections, glomeruli were contoured, and threshold intensity of each channel was measured as previously. Colocalization of the thresholded images in kidney sections was determined using the JACoP plugin in ImageJ.

For transmission electron microscopy, kidney sections were fixed with 2.5% glutaraldehyde in 0.1 M sodium cacodylate buffer and processed at the McGill University Facility for Electron Microscopy Research (Navarro‐Betancourt et al., 2020). Tissues were imaged with a Tecnai 12 electron microscope linked to an AMTV601 CCD camera (Field Electron and Ion Company, Hillsboro, OR). Quantitative analysis of electron micrographs, including foot process and GBM width was performed using Image J, as described previously (Cybulsky et al., 2018; Navarro‐Betancourt et al., 2020).

### 2.3 SLK KO GECs

Production of these cells was described in detail previously (Woychyshyn et al., 2020). Briefly, glomeruli were isolated from SLK^flox/flox^; +/+ mice. Glomeruli were suspended in K1 medium and were added to type I collagen‐coated plates. The plates were incubated at 37°C until cells started to grow outside of the glomeruli. Cells were immortalized with a temperature‐sensitive SV40 lentivirus. Clones of GECs were individually expanded at 33°C and lysates were analyzed by immunoblotting for markers of podocyte differentiation. A clone of GECs demonstrating the presence of all podocyte markers was chosen for the transduction with the tamoxifen‐inducible Cre recombinase and mCherry. Transduced cells were incubated with tamoxifen and the cells expressing a high level of mCherry, which correlates with high expression of Cre recombinase were selected by cell sorting. Deletion of SLK in the GECs was verified by PCR and immunoblotting (Woychyshyn et al., 2020). Untransduced cells were the control GEC line.

For experiments, GECs were allowed to proliferate for 1 day at 33°C, then switched to 37°C to differentiate for 24 h. Selected wells were incubated with adriamycin for 24 h. After incubation, cells were fixed for IF microscopy, or lysates were prepared for immunoblotting. The cell adhesion assay was described previously (Cybulsky et al., 2018).

### 2.4 Visualization of FACs

To visualize FACs, GECs were fixed with 4% paraformaldehyde (37°C), permeabilized with 0.5% Triton X‐100 and blocked with 3% BSA. Alternatively, GECs were fixed with ice‐cold methanol. Cells were stained with antibodies against FAC proteins for 24 h (4°C) followed by secondary antibodies and Hoechst H33342 (to stain nuclei), as described previously (Chung et al., 2019, 2023; Kachurina et al., 2016). Z‐stack images were acquired on a Zeiss Axio Observer inverted fluorescence microscope with visual output connected to an AxioCam MRm monochrome camera (Carl Zeiss AG; Toronto, ON). IF intensities and cell measurements were performed using ImageJ, as described previously (Chung et al., 2019; Horzum et al., 2014; Kachurina et al., 2016). ImageJ allows pre‐definition of particle size and the threshold of IF intensity to count FACs and estimate cell size. Color photographs were split into three separate channels and converted to grayscale. Cell contours were selected using the freehand tool on grayscale images, and the area of the cross‐section was measured. Image histogram plots were obtained, representing the range of pixel brightness values in each image. Threshold intensity was set at the end of the histogram declining slope (thereby only including the brightest fluorescent signals and omitting cellular features and background). Previous studies of FACs demonstrated that most FAC protein particles are between 0.2 and 10 μm^2^ (Horzum et al., 2014). We initially analyzed particles between 0.2 and 2 μm^2^, 2 and 4 μm^2^, 4 and 6 μm^2^, 6 and 8 μm^2^, and 8 and 10 μm^2^ for paxillin, vinculin and talin. In our study, almost all particles were found between 0.2 and 4 μm^2^, with the large majority found between 0.2 and 2 μm^2^ for the three proteins. Hence, we were able to divide FACs into small (0.2–2 μm^2^) and large (2–4 μm^2^) particles. The number of particles and the total cross‐sectional area of the particles whose areas fell within this set range were measured. Results were normalized per 1000 μm^2^ of cell area. Colocalization of the thresholded images was determined using the JACoP plugin in ImageJ.

### 2.5 Immunoblotting

The protocol for immunoblotting was described earlier (Navarro‐Betancourt et al., 2020; Woychyshyn et al., 2020). Chemiluminescence was detected in a ChemiDoc Touch Imaging System (Bio‐Rad; Mississauga, ON); signal saturation was monitored with Image Lab (Bio‐Rad) and only signal intensities within a linear range were analyzed. Band densitometries were quantified using ImageJ and values were normalized to the expression of β‐actin.

### 2.6 Mass spectrometry

Culture and transfection of COS1 cells (African green monkey kidney) was carried out as described previously (Cybulsky et al., 2017). Lysates of COS1 cells or GECs were subjected to SDS‐PAGE. Cell lysates underwent in‐gel trypsin digestion; only the top portion of the gel was used for talin analysis in COS1 cells. This was followed by high‐performance liquid chromatography and tandem mass spectrometry using a Thermo Orbitrap Fusion LC–MS/MC (Thermo‐Fisher Scientific), as described previously (Navarro‐Betancourt et al., 2022). Proteins were identified using the Mascot 2.5.1 search engine (Matrix Science, Boston, MA) and spectral counts were analyzed with Scaffold_5 software (Proteome Software, Portland, OR).

### 2.7 Dataset analyses

To determine potential SLK phosphorylation sites in proteins, as well as protein kinases that might phosphorylate focal adhesion proteins, we used a database encompassing synthetic peptide libraries that profiled the substrate sequence specificity of 303 serine/threonine kinases (Johnson et al., 2023). Potential phosphorylation sites were further verified using a database of proteomic discovery mass spectrometry (PhosphositePlus) (Hornbeck et al., 2015), as well as a phosphoproteomic atlas of nine mouse tissues (Huttlin et al., 2010) and a phosphoproteomic analysis of mouse glomerular proteins (Rinschen et al., 2014).

### 2.8 Statistics

In all experiments, data are presented as mean ± standard deviation of the mean. The Student's *t*‐test was used to determine significant differences between two groups. In experiments with two groups and multiple treatments or time‐points, two‐way ANOVA was used to determine significant differences among groups; where relevant, additional comparisons were calculated and values were adjusted according to the Bonferroni method. Statistical analyses and graphical presentation of results were performed using GraphPad Prism software. Significant differences between two groups are displayed with lines between columns (*p*‐values are presented above the lines). In the absence of such lines, differences were not statistically significant.

## 3 RESULTS

### 3.1 SLK deletion in podocytes exacerbates podocyte injury in adriamycin nephrosis

**FIGURE 1:**
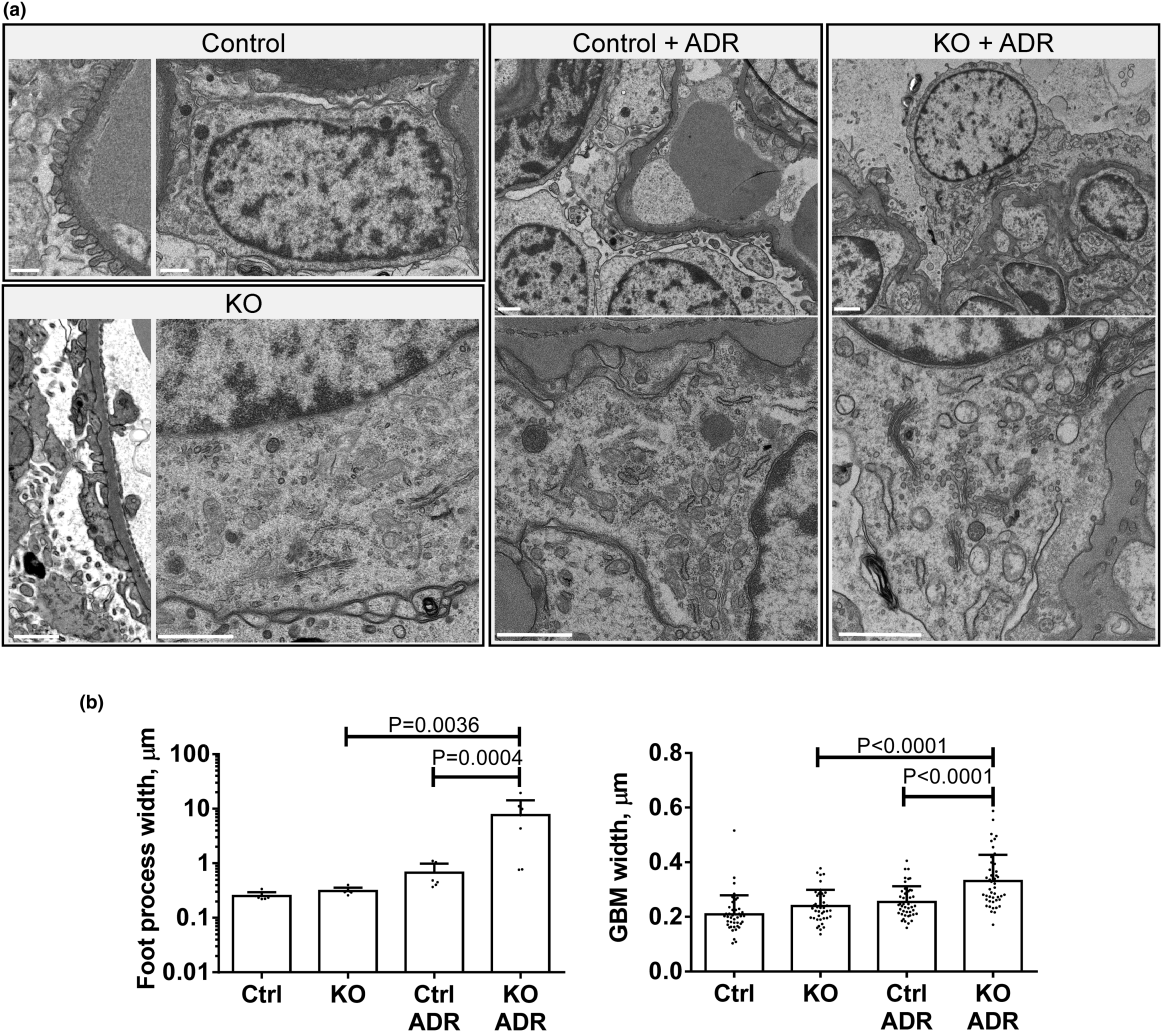
Deletion of SLK in podocytes exacerbates ultrastructural podocyte injury in adriamycin nephrosis. (a) Control mice show normal podocyte foot processes. Podocyte‐specific SLK KO mice show slight focal foot process effacement. Both groups of mice show normal organelles. Adriamycin (ADR) has a minimal effect on foot process effacement and structure of organelles in control mice, but induces foot process effacement, glomerular basement membrane (GBM) widening, microvesiculation of podocyte plasma membranes, as well as swelling of the endoplasmic reticulum and mitochondria, and loss of mitochondrial cristae in SLK KO mice. (b) Quantification of foot process and GBM width (foot process width consists of one measurement per glomerulus and GBM width is five measurements per glomerulus in two mice per group, 3–4 glomeruli/mouse. Bars = 1.0 μm. KO, knockout.

Mice with podocyte‐specific deletion of SLK develop albuminuria, podocyte loss and focal foot process effacement as they age (Cybulsky et al., 2018). In mice with adriamycin nephrosis (experimental FSGS, induced at 3–4‐months and followed for 4 weeks), SLK deletion exacerbates albuminuria (~5‐fold greater in SLK KO mice compared to control), and loss of podocytes (Woychyshyn et al., 2020). We examined podocyte ultrastructure in these young mice with adriamycin nephrosis (Figure 1a). Untreated SLK KO mice showed mild podocyte foot process effacement and microvillous plasma membrane transformation compared to control. Adriamycin induced mild focal foot process effacement in control mice, and this was exaggerated markedly in SLK KO mice. Foot process width, as well as GBM width in adriamycin‐treated SLK KO mice were significantly greater compared to adriamycin‐treated control and untreated SLK KO (Figure 1b). Furthermore, adriamycin‐treated SLK KO mice showed some damage of organelles, including swelling of mitochondria and endoplasmic reticulum. We did not observe areas of podocyte detachment from the GBM.

### 3.2 SLK reduces GEC adhesion

**FIGURE 2:**
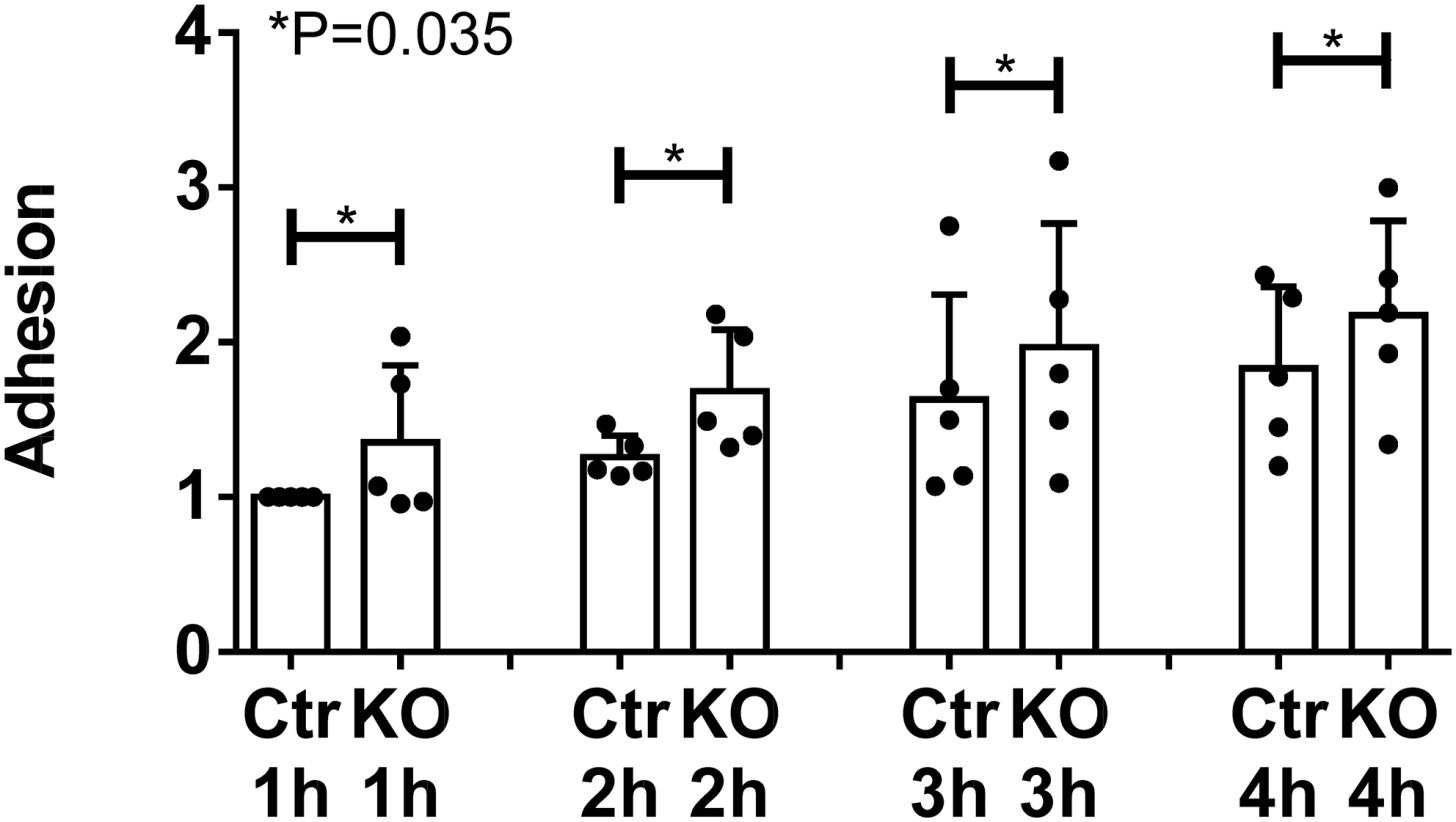
SLK KO glomerular epithelial cells (GECs) show increased adhesion compared to control (Ctr). GECs were placed into suspension and were then plated into collagen‐coated tissue culture wells. Adhesion was measured after 1–4 h. Values are normalized to the Ctr, 1 h group. *p* < 0.035 Ctr versus KO (two‐way ANOVA incorporating the four time‐points), five experiments. KO, knockout.

It is believed that in glomerulopathies, podocyte foot process effacement may be associated with enhanced adhesion to the GBM (Kriz et al., 2013), and podocytes may spread to cover denuded GBM and counteract detachment. Given that SLK was shown to be involved in modulating adhesion of cultured cells (Al‐Zahrani et al., 2013; Garland et al., 2021), our observations in adriamycin nephrosis prompted further study of adhesion in podocytes. We show that compared to control GECs, adhesion to collagen increases in SLK KO GECs (Figure 2). This result is consistent with our earlier study, where knockdown of SLK in rat GECs with siRNAs increased adhesion to collagen (Cybulsky et al., 2018).

### 3.3 Candidate SLK phosphorylation sites in adhesion proteins

Next, we investigated adhesion‐associated proteins as potential substrates of SLK by analyzing a database of peptides with serine and threonine sites phosphorylated by SLK (Johnson et al., 2023). Using a cutoff at the 90th percentile (peptide motifs with the highest probability of being SLK phosphorylation motifs), potential SLK phosphorylation sites were identified in 4352 proteins (Table S1). Interestingly, the top‐ranked SLK phosphorylation site is T567 in ezrin (Johnson et al., 2023), which we and others have previously characterized biochemically (Cybulsky et al., 2017; Viswanatha et al., 2012). Among the proteins potentially phosphorylated by SLK, 236 and 92 were associated with focal adhesion genes found in the GO:0005925 and KEGG datasets, respectively (Table S1). These results suggest that SLK can potentially phosphorylate substrates that are involved in the regulation of cell adhesion.

From among these potential SLK substrate proteins, we then selected three for further study, including paxillin, talin and vinculin. These three proteins are well‐defined components of FACs, and paxillin and talin were reported to be SLK substrates in fibroblasts (Quizi et al., 2013) and Drosophila muscle cells (Katzemich et al., 2019), respectively. Furthermore, talin and vinculin are important for preservation of podocyte structure (Lausecker et al., 2018; Tian et al., 2014). Paxillin was previously reported to be phosphorylated by SLK at S250 in fibroblasts (Figure S1) (Quizi et al., 2013), but surprisingly, in the phosphopeptide database, the paxillin S250 site was identified as a SLK phosphorylation site at only the 66th percentile (Johnson et al., 2023). Other potential sites of SLK phosphorylation (>90th percentile) in paxillin are S90, S119 and S533 (Figure S1). The fly SLK ortholog Slik phosphorylates talin at T152 (Katzemich et al., 2019). This site is highly conserved and corresponds to human or mouse talin T144 (Goult et al., 2010), which was reported to be phosphorylated in humans (Li et al., 2016). In the phosphopeptide database, T144 is a high probability potential site of SLK phosphorylation, as are talin T150, T354, S405, and T430 (Figure S1). Two potential SLK phosphorylation sites were identified in vinculin (S383 and T793; Figure S1).

The potential SLK phosphorylation sites and a consensus sequence (Figure S1) are based on experiments involving peptide phosphorylation by recombinant kinases (Johnson et al., 2023), which may not represent true phosphorylation sites in intact proteins or in the context of kinases in intact cells. Nevertheless, we found that all of the above putative phosphorylation sites in the three proteins (except one vinculin site) are indeed phosphorylated in intact proteins in proteomic discovery mass spectrometry studies (PhosphositePlus) (Hornbeck et al., 2015). However, we found that only talin S405 and paxillin S533 are described in a phosphoproteomic characterization of nine mouse tissues (i.e., constitutive phosphorylation) (Huttlin et al., 2010), and none of the sites in the three proteins are described in a phosphoproteomic analysis of mouse glomerular proteins (Rinschen et al., 2014).

### 3.4 Effect of SLK on the composition of FACs

The above dataset analysis provided a basis for further experimental investigation of paxillin, vinculin and talin in FACs. We evaluated these FAC components in control and SLK KO GECs that were untreated or treated with adriamycin. Proteins were quantified using IF microscopy by setting an appropriate FAC particle size and threshold for counting in ImageJ (Horzum et al., 2014). We used particle sizes of 0.2–2 μm^2^ and 2–4 μm^2^ for small and large FACs, respectively. It is believed that large focal adhesions bind more strongly to the extracellular matrix and transduce force on stress fibers, and may therefore be more relevant for cell anchorage, while smaller adhesions are short‐lived and mediate traction forces for cell migration (although other factors may also influence cell adhesion and migration parameters) (Elineni & Gallant, 2011; Gallant et al., 2005; Kim & Wirtz, 2013). We calculated both the numbers of FACs (particles) and total FAC (particle) area, and report significant differences that were present in parallel in both parameters.

**FIGURE 3:**
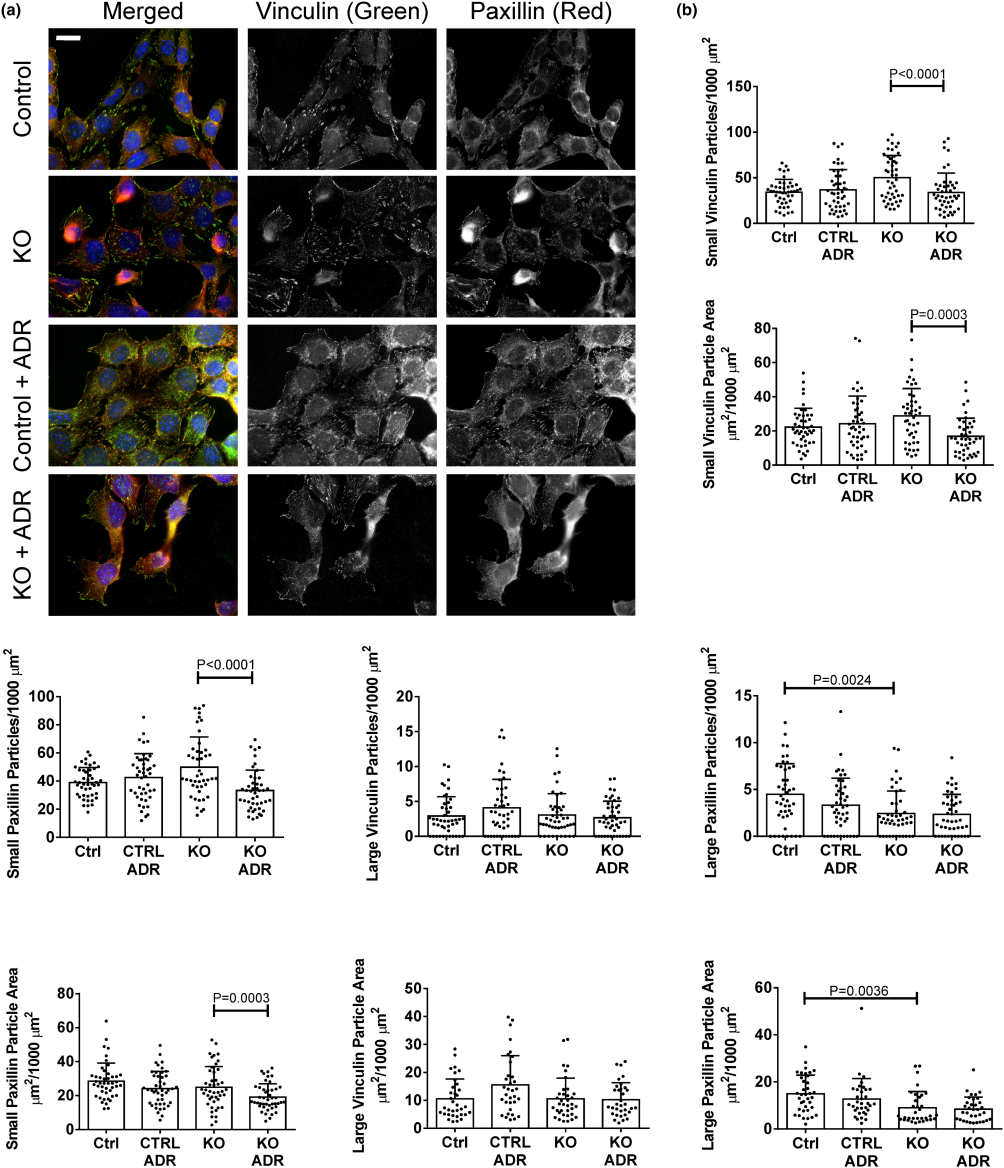
Effect of SLK on vinculin and paxillin in glomerular epithelial cells (GECs). Control or SLK KO GECs were untreated or incubated with adriamycin (ADR; 0.5 μM) for 24 h. Cells were fixed and stained with antibodies to vinculin and paxillin. (a) Representative immunofluorescence (IF) micrographs. (b) Quantification of the numbers of small and large particles (FACs) and particle areas (Materials and Methods). Measurements were done in 20 cells per group in three experiments. Bar = 20 μm. KO, knockout.

**FIGURE 4:**
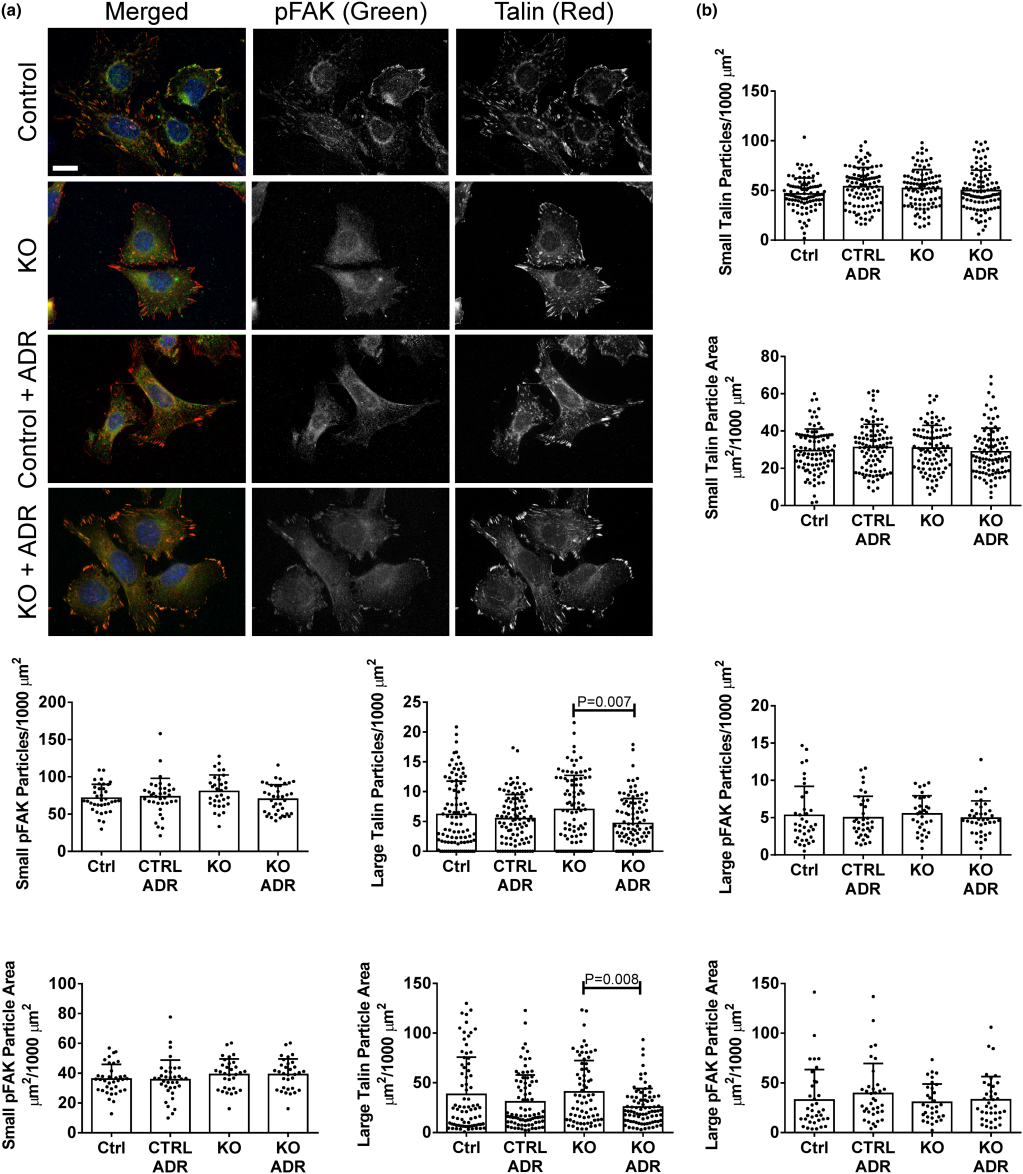
Effect of SLK on talin and phospho‐Y397 FAK (pFAK) in glomerular epithelial cells (GECs) (see legend to Figure 3). Measurements were done in 52–58 cells per group in five experiments (talin) and 32–38 cells per group in two experiments (pFAK). Bar = 20 μm.

In untreated control GECs, the numbers and total adhesion areas of small vinculin (Figure 3), paxillin (Figure 3) and talin FACs (Figure 4) were comparable among groups. The numbers of large vinculin, paxillin and talin FACs were lower compared to the small FACs, as were the large vinculin and paxillin adhesion areas, while the large talin area was comparable to the small talin area, despite fewer large particles. Deletion of SLK did not alter the numbers or areas of small vinculin, paxillin and talin particles, although adriamycin reduced the numbers and areas of small vinculin and paxillin particles in SLK KO GECs (Figure 3). Deletion of SLK reduced the number and area of large paxillin particles, and there was no additional effect of adriamycin. In contrast, large talin particle numbers and areas were reduced by adriamycin only in SLK KO GECs (Figure 4). Thus, deletion of SLK resulted in loss of small paxillin and vinculin FACs, as well as large paxillin and talin FACs following adriamycin‐induced injury (and in one case under basal conditions). Total cellular levels of vinculin, paxillin and talin in GECs were measured by immunoblotting. There were no apparent difference among control and SLK KO GECs, untreated or treated with adriamycin (Figure S2).

To determine if the SLK‐mediated changes in FAC proteins were associated with FAC stability versus turnover, we monitored the amount of phospho‐Y397 FAK (pFAK) in FACs (Lausecker et al., 2018). No significant changes were observed in small and large pFAK particle numbers or areas among the four groups (Figure 4). Since talin and pFAK particles were measured together, we calculated the Pearson correlation coefficient between pFAK and talin (Figure S3a). In untreated control cells, the value was ~0.6, suggesting that a portion of pFAK was found in the same FACs as talin, and a portion in non‐talin FACs. There were no significant differences in the Pearson correlation coefficient among the four groups of cells. It should also be noted that the Pearson correlation coefficient for colocalization of vinculin and paxillin was ~0.75 in the four groups of cells shown in Figure 3, and was not signifcantly different among groups (Figure S3b).

### 3.5 Substrate phosphorylation by SLK

**FIGURE 5:**
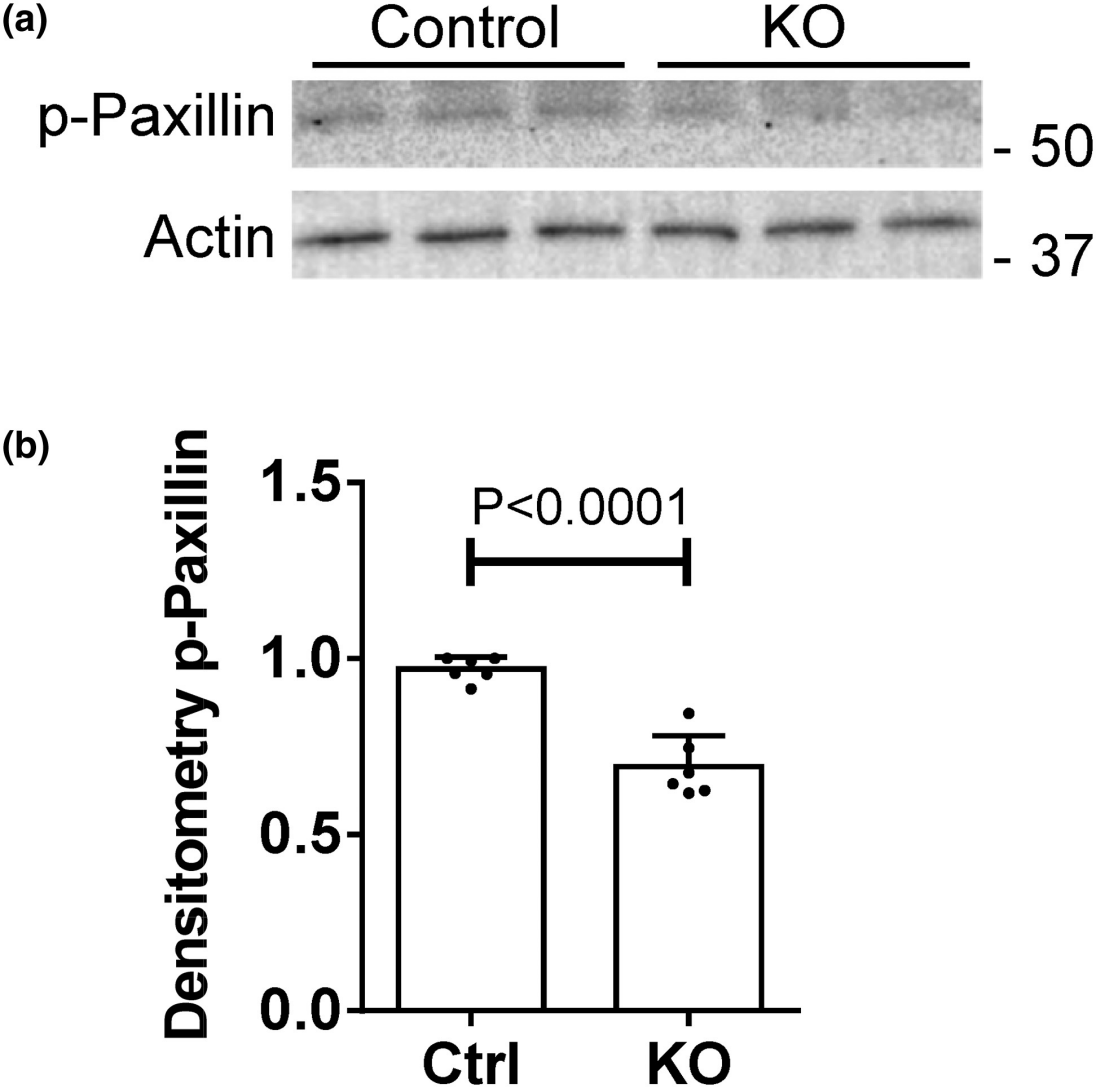
SLK phosphorylates paxillin. (a) Lysates of control and SLK KO glomerular epithelial cells (GECs) were immunoblotted with antibody to paxillin S250 (p‐Paxillin). (a) Representative immunoblot. (b) Densitometric quantification of two experiments performed in triplicate. Densitometry of p‐Paxillin is normalized to actin. KO, knockout.

Next, we examined if paxillin or talin were phosphorylated by SLK. In resting epithelial cells, SLK is believed to be constitutively active, and phosphorylation of ezrin by SLK was described as a “phosphocycling” process (Viswanatha et al., 2012). Thus, we would expect that if paxillin is a substrate of SLK in GECs, then S250 phosphorylation should be detectable in resting cells. We compared paxillin S250 phosphorylation in control and SLK KO GECs by immunoblotting with a phospho‐S250‐specific antibody (Quizi et al., 2013). Phospho‐paxillin was apparent in both control and SLK KO GECs, with the signal being ~40% greater in control GECs (Figure 5). The signal in SLK KO GECs may reflect basal paxillin phosphorylation by another protein kinase. Indeed, based on analysis of the paxillin S250 peptide phosphorylation (percentile score >95th), there are several candidate protein kinases that can phosphorylate paxillin at S250, including PRKD1/2, BRSK1/2, DLK, CAMK1A, PKCG, and PKCB (Johnson et al., 2023).

To examine if SLK can phosphorylate talin at T144, we first overexpressed the kinase domain of SLK (SLK 1–373) in COS1 cells by transfection of HA‐SLK 1–373 cDNA (or GFP plasmid in control; Figure S4) (Cybulsky et al., 2017). Cell lysates were then subjected to mass spectrometry analysis (Navarro‐Betancourt et al., 2022). T144 is found within the talin 139–146 tryptic peptide (Figure S1). In two experiments, the talin 139–146 peptide was found in both control and SLK‐transfected COS1 cells; however, T144 in the peptide was not phosphorylated under either condition. We also examined T144 phosphorylation by mass spectrometry in a dataset we generated earlier in resting SLK‐replete mouse GECs (Navarro‐Betancourt et al., 2022). Similarly to COS1 cells, the talin 139–146 peptide was found in duplicate samples of resting GECs, but T144 was not phosphorylated. Therefore, we conclude that even though the analogous talin site was phosphorylated by Drosophila Slik, talin phosphorylation by SLK may not occur in mammalian systems, or occurs only with low stoichiometry.

Among the other putative SLK phosphorylation sites in talin (T150, T354, S405, and T430) (Figure S1) (Johnson et al., 2023), peptides containing T354 were observed by mass spectrometry in one SLK‐transfected COS1 cell sample and in SLK‐replete mouse GECs (Navarro‐Betancourt et al., 2022), while peptides containing T430 were observed in one control COS1 cell sample and in mouse GECs. None of these peptides showed phosphorylation. Peptides containing T150 and S405 sites were not found in COS1 cells or in GECs. We also interrogated our mass spectrometry analysis of resting SLK‐replete mouse GECs for putative paxillin and vinculin SLK phosphorylation sites (Figure S1). Peptides containing the paxillin S90 and S119 putative phosphorylation sites were present in four samples, but none showed phosphorylation. Peptides containing paxillin S250 and S533 were not found among six samples. Peptides containing vinculin S383 and T793 putative phosphorylation sites were not found among eight samples.

### 3.6 Effect of SLK on talin and vinculin in vivo

**FIGURE 6:**
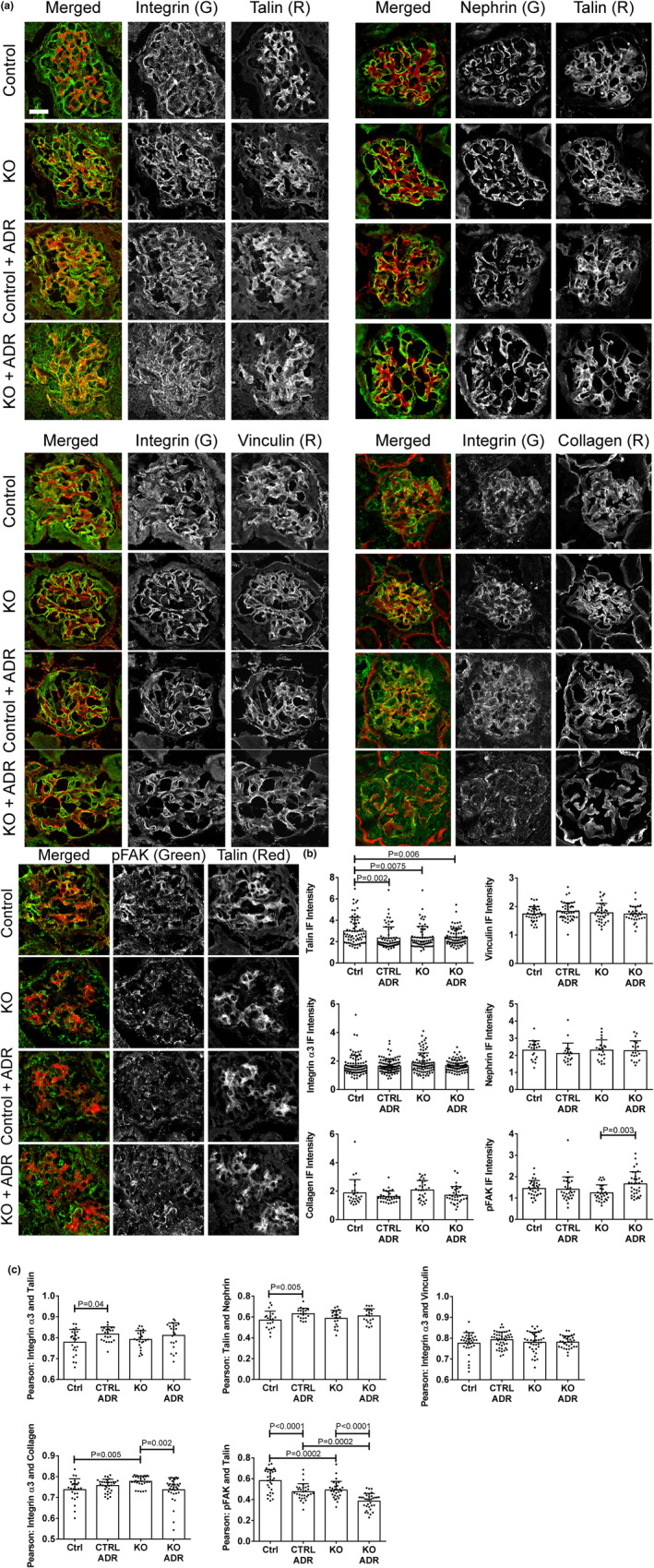
Effect of SLK on glomerular FAC proteins. (a) Kidney sections from control and SLK KO mice that were untreated or treated with adriamycin were stained with antibodies, as indicated. Representative images are presented. (b) Quantification of glomerular immunofluorescence (IF) intensity. Talin: 13 mice/group, 4–9 measurements/mouse; vinculin: 5–7 mice/group, 3–10 measurements/mouse; α3‐integrin: 14 mice/group, 2–10 measurements/mouse; nephrin: 5 mice/group, 5–6 measurements/mouse; α5‐collagen IV: 5–7 mice/group, 2–10 measurements/mouse; pFAK: 4 mice/group, 7–9 measurements/mouse. (c) Pearson correlation coefficients. α3‐integrin and talin: 4 mice/group, 5–7 measurements/mouse; nephrin and talin: 5 mice/group, 5–6 measurements/mouse; α3‐integrin and vinculin: 5–7 mice/group, 3–10 measurements/mouse; α3‐integrin and α5‐collagen IV: 5–7 mice/group, 2–10 measurements/mouse; pFAK and talin: 4 mice/group, 7–9 measurements/mouse. KO, knockout.

**FIGURE 7:**
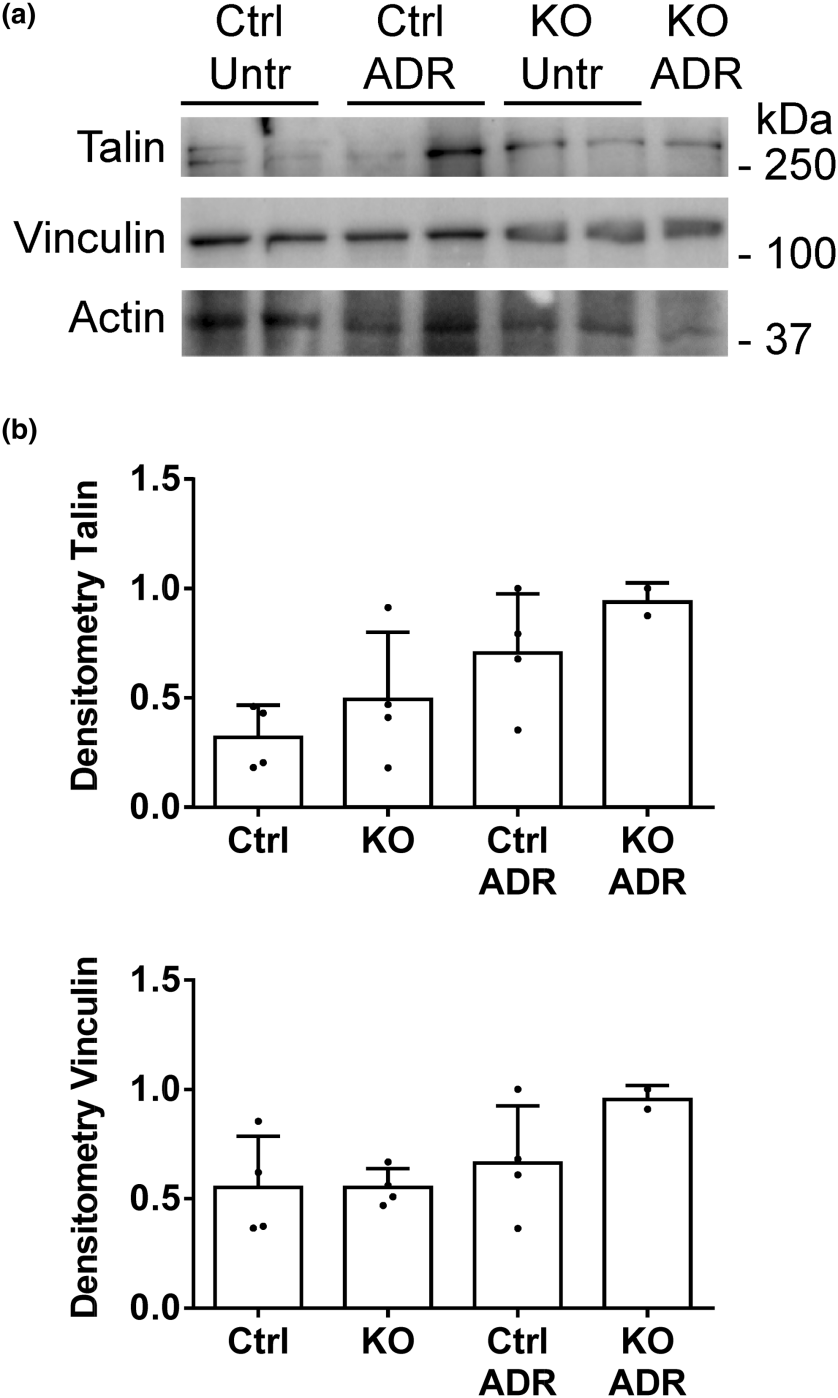
Effect of SLK on expression of glomerular talin and vinculin. (a) Glomerular lysates were immunoblotted with antibodies to talin and vinculin. (a) Representative immunoblot. (b) Densitometric quantification. There are no significant differences among groups. There are four kidneys per group except for two kidneys in KO adriamycin (ADR). Ctrl, control. KO, knockout. Densitometry of talin and vinculin is normalized to actin.

In vivo, podocytes are attached to the GBM via integrins, which in turn, are anchored to the actin cytoskeleton through proteins forming structures that may be analogous to FACs in cultured cells. We undertook to examine the localization of talin and vinculin in the glomerulus in relation to proteins at the podocyte basal adhesive surface and slit diaphragm, using IF microscopy (Lausecker et al., 2018). We could not address paxillin in these experiments, since IF paxillin staining in the glomerulus was weak. Kidney sections from control and SLK KO mice that were untreated or treated with adriamycin were co‐stained with antibodies to talin or vinculin together with markers of the podocyte basal surface (α3‐integrin) (Kreidberg et al., 1996) or slit diaphragm (nephrin). Compared to untreated control mice, global glomerular talin IF intensity was ~25% lower in SLK KO mice, as well as in the adriamycin‐treated groups (Figure 6a,b). However, there were no significant differences in talin expression when monitored by immunoblotting (Figure 7). Also, there were no significant differences in the expression of vinculin (IF intensity and immunoblotting), as well as α3‐integrin or nephrin (IF intensities) among groups (Figures 6a,b and Figure 7). The Pearson correlation coefficients for talin and α3‐integrin IF signals, as well as vinculin and α3‐integrin were ~0.8, and the coefficient was ~0.6 for talin and nephrin, implying predominant localization of talin and vinculin in podocytes (Figure 6c). Colocalization of talin and α3‐integrin, as well as talin and nephrin were comparable among groups, with no statistically significant differences between untreated control and untreated KO mice, although there was a slight statistically significant increase in adriamycin‐treated control mice compared to untreated control (Figure 6c). Colocalization of vinculin and α3‐integrin did not differ among groups (Figure 6c). Thus, there were only minor effects of SLK deletion on the colocalization of talin and vinculin with proteins at the basal aspect of podocytes or the slit diaphragm.

We also co‐stained kidney sections with antibodies to α3‐integrin and α5‐collagen IV, a component of the GBM. There were no differences in α5‐collagen IV intensities among groups (Figure 6a,b) and the Pearson correlation coefficients for α3‐integrin and α5‐collagen IV were ~0.75 (Figure 6c). Colocalization of α3‐integrin and α5‐collagen IV was increased slightly in untreated SLK KO mice compared to untreated control, but there were no significant effects of adriamycin (Figure 6c). The result suggests that despite ultrastructural changes, including marked foot process effacement in SLK KO mice with adriamycin nephrosis (Figure 1), the interaction of integrins at the podocyte basal edge with the GBM (podocyte‐GBM interface) was not altered substantially by SLK activity or adriamycin. This result is in keeping with absence of significant podocyte detachment, as observed by electron microscopy (Figure 1).

Finally, we examined glomerular pFAK using IF microscopy (Ma et al., 2010) (we did not detect pFAK reliably by immunoblotting). pFAK intensity was 15%–20% greater in adriamycin‐treated SLK KO mice (Figure 6a,b). Interestingly, colocalization of pFAK and talin was reduced significantly in all groups of mice compared with untreated control, with the greatest reduction in adriamycin‐treated SLK KO mice (Figure 6c). This result may reflect a shift in pFAK away from talin FAC‐like structures.

## 4 DISCUSSION

In the present study, we show that deletion of SLK in podocytes exacerbates podocyte foot process effacement in young mice with experimental FSGS (adriamycin nephrosis), consistent with development of albuminuria, shown previously (Woychyshyn et al., 2020). Deletion of SLK in aging mice also leads to podocyte injury (Cybulsky et al., 2018). We and others demonstrated that SLK can phosphorylate/activate ezrin (Cybulsky et al., 2017), and deletion of SLK reduces the colocalization of ezrin and podocalyxin in the glomerulus (Woychyshyn et al., 2020). Other studies demonstrated that phosphorylation of ezrin was essential to maintain the association of podocalyxin with F‐actin at the apical surface of podocyte foot processes (Al‐Momany et al., 2014; Takeda et al., 2001), and that deletion of podocalyxin in podocytes results in proteinuria (Refaeli et al., 2020). Based on these results, it is reasonable to conclude that SLK can regulate podocyte structural integrity, at least in part, by maintaining the apical structure of foot processes; however, whether ezrin is essential to the maintenance of the apical structure remains unclear (Hatano et al., 2018). Thus, an important question is whether podocyte adhesion to the GBM under basal conditions or in adriamycin nephrosis is dependent on the interaction of SLK with proteins in FACs.

We focused on three potential SLK substrates in FACs, including paxillin, talin and vinculin. An analysis of phosphopeptide sites phosphorylated by SLK suggested that these proteins may be candidate SLK substrates. Paxillin was previously shown to be a substrate of SLK in fibroblasts (Quizi et al., 2013), while talin was a substrate of the Drosophila SLK homolog Slik (Katzemich et al., 2019), and talin was shown to be a critical component of adhesion in podocytes (Tian et al., 2014). In addition, we examined vinculin, which plays a role in podocyte adhesion, although it had not been shown to be a SLK substrate (Lausecker et al., 2018; Tian et al., 2014).

In GECs, deletion of SLK resulted in reduction of small paxillin and vinculin FACs in the context of adriamycin‐induced injury. As small FACs are believed to mediate traction forces for cell migration (Kim & Wirtz, 2013), the result is in keeping with our earlier observation that loss of SLK reduces GEC migration (Cybulsky et al., 2017). Deletion of SLK also reduced large paxillin particles, while large talin particles were reduced in SLK KO GECs following adriamycin treatment. Large FACs are believed to mediate cell anchorage to substrata, which would imply that deletion of SLK could potentially reduce cell adhesion, but this was not observed (see below). SLK did not affect Y397 FAK phosphorylation in GECs, suggesting that FACs were stable with no significant turnover.

Interestingly, compared to control cells, adhesion of GECs to collagen increased modestly in SLK KO GECs, in keeping with an earlier study where we showed that siRNA knockdown of SLK increased GEC adhesion (Cybulsky et al., 2018). This also parallels podocyte foot process effacement in SLK KO mice, and is in keeping with the view that foot process effacement may be associated with enhanced adhesion to the GBM (Kriz et al., 2013). The anti‐adhesive effect of SLK may have been mediated through a non‐FAC pathway. For example, besides integrins, transmembrane heparan sulfate proteoglycan receptors, such as the syndecan family, may mediate cell‐matrix interactions, and may be functionally important in podocytes (Lennon et al., 2014; Sachs & Sonnenberg, 2013). Dystroglycans are cell surface adhesion receptors that link matrix proteins to the actin cytoskeleton. These molecules are found in podocytes, although podocyte‐specific deletion of dystroglycan in mice caused only mild GBM thickening, suggesting that dystroglycan is not an important adhesion receptor in podocytes (Lennon et al., 2014; Sachs & Sonnenberg, 2013). Type XVII collagen is another podocyte adhesion molecule (Sachs & Sonnenberg, 2013). It should also be noted that KO of talin in cultured GECs caused only a modest reduction in cell adhesion (Tian et al., 2014), suggesting that there are redundancies in adhesion pathways. The anti‐adhesive effects of SLK will require further study.

While analysis of phosphopeptide sites phosphorylated by SLK suggested that FAC proteins may be candidate SLK substrates, we did not observe SLK‐dependent phosphorylation of talin at T144 in COS1 cells and GECs. Possibly, talin phosphorylation is specific to Slik in Drosophila (Katzemich et al., 2019). In SLK‐replete GECs, paxillin S250 phosphorylation was increased modestly above a significant basal level in SLK KO GECs. In fibroblasts, S250 phosphorylation facilitated migration and FAC turnover, but did not affect adhesion (Quizi et al., 2013). The physiologic role of the minor SLK‐dependent paxillin phosphorylation in GECs is unclear, given that SLK KO did not significantly affect FAC turnover. Indirect phosphorylation of adhesion proteins by SLK is also a possibility to consider, e.g., polo‐like kinase‐1 is a SLK substrate (Cybulsky et al., 2016; Garland et al., 2021), and paxillin might be phosphorylated at S143 via SLK‐dependent activation of polo‐like kinase‐1 (Johnson et al., 2023). Definition of additional substrates of SLK will require further study.

Many studies into the function of FACs, integrins and cell adhesion have been performed in cultured cells, including GECs. The relation of GEC culture findings to podocyte adhesion to the GBM in vivo is not straightforward. Nevertheless, in vivo deletion of podocyte integrins and talin induce marked phenotypic changes (Lennon et al., 2014). We showed that glomerular talin IF intensity was reduced by ~25% in SLK KO mice, as well as in the adriamycin‐treated groups, compared to untreated control mice, although such differences were not evident by immunoblotting. These distinct results might be related to differences in techniques. Further studies will be required to explain these findings, although the results on glomerular talin expression by immunoblotting were consistent with results in cultured GECs. There were no differences observed in glomerular vinculin, α3‐integrin or nephrin intensities among groups. Talin and vinculin were extensively colocalized with α3‐integrin, and talin was also colocalized with nephrin. There were no major differences in colocalization among control and SLK KO mice that were untreated or received adriamycin. The colocalization of integrins with GBM collagen was not altered substantially by SLK deletion or adriamycin. These results imply that the podocyte‐GBM interface was probably not affected by SLK deletion or disease. Whether a decrease in glomerular talin could be functionally significant in impairing podocyte adhesion to the GBM is unclear. As noted above, KO of talin in GECs does not affect adhesion substantially (Tian et al., 2014), and furthermore, heterozygous talin KO mice (50% talin expression) did not show phenotypic abnormalities (Monkley et al., 2000). Nevertheless, given that podocyte‐specific talin KO mice show proteinuria and foot process effacement (Tian et al., 2014), a reduction in talin could potentially contribute to proteinuria and foot process effacement in SLK KO mice with adriamycin nephrosis.

pFAK intensity increased modestly in adriamycin‐treated SLK KO mice, in keeping with an earlier observation that in glomerular injury there was increased pFAK in podocytes, associated with proteinuria and foot process effacement (Ma et al., 2010). It was suggested that increased pFAK may represent an injury response, since podocyte‐specific deletion of FAK in mice protected mice from proteinuria and podocyte injury in experimental nephritis (Ma et al., 2010). Furthermore, in our study, loss of SLK in the context of adriamycin reduced colocalization of pFAK and talin. This result suggests that pFAK was increased in areas that were relatively depleted of talin, although the significance of this observation will require further study.

The resolution of light/confocal IF microscopy is ~200 nm, while electron microscopy provides resolution that is up to 1000‐fold greater. Thus, IF microscopy is not sufficiently sensitive to resolve detailed changes in foot process structure, although the IF staining pattern could potentially be disrupted by significant podocyte detachment (which we did not observe). By immunoelectron microscopy, talin has been localized at the base of podocyte foot processes and adjacent to the slit diaphragms (Tian et al., 2014), although to our knowledge there are no results available in diseases. Insights into subcellular relocalization of podocyte proteins in glomerulopathies have been provided by studies into nephrin. Anti‐nephrin antibodies labeled the interpodocyte areas of normal rat glomeruli at the level of the filtration slit diaphragms. In the diseased glomerulus, nephrin was observed along the apical plasma membrane of podocytes, but most of the label was still found at the filtration slits (Luimula et al., 2000). Quantitative ultrastructural studies of puromycin aminonucleoside nephrosis in rats showed that nephrin expression was altered in the areas that showed foot process effacement. However, nephrin expression was comparable with that in control animals in areas where the foot process interspaces were preserved (Lee et al., 2004). In humans with glomerulopathies, nephrin labeling appeared to be reduced in areas of foot process effacement (Huh et al., 2002). Super resolution imaging in adriamycin nephrosis showed loss of nephrin near the GBM, and redistribution to the podocyte cytoplasm and apical surface. In contrast, CD2AP (a protein that normally associates with nephrin at the slit diaphragm) redistributed adjacent to the GBM (Suleiman et al., 2017). In the future, analogous approaches could be applied to examine FAC proteins and integrins.

In summary, while previous studies have provided support for SLK‐mediated ezrin phosphorylation and a SLK‐ezrin‐F‐actin axis in maintaining apical podocyte foot process structure, the present study has not implicated direct effects of SLK on FAC proteins at the basal aspect of podocytes. However, some changes in these proteins were evident in SLK KO GECs and mice, particularly in the context of adriamycin. A recent study has proposed ezrin linking F‐actin with ephrin B1, and ephrin B1 interacting with nephrin (Fukusumi et al., 2021). This pathway involving ezrin could potentially be regulated by SLK. Finally, ultrastructural damage to podocyte organelles in SLK KO mice with adriamycin nephrosis suggests that SLK could play a protective role in preserving organelle function. These non‐adhesion pathways can be examined in future studies.

## AUTHOR CONTRIBUTIONS

Andrey V. Cybulsky conceived and designed the research. Joan Papillon, Craig Bryan, and José R. Navarro‐Betancourt performed experiments. Luc A. Sabourin generated reagents. Andrey V. Cybulsky analyzed the data and interpreted results of experiments. Andrey V. Cybulsky wrote the manuscript. All authors reviewed and approved the manuscript.

## FUNDING INFORMATION

This work was supported by Research Grants from the Canadian Institutes of Health Research (PJ9‐166216 and PJ9‐169678) and the Kidney Foundation of Canada, and the Catherine McLaughlin Hakim Chair.

## CONFLICT OF INTEREST STATEMENT

The authors declare that the research was conducted in the absence of any commercial or financial relationships that could be construed as a potential conflict of interest.

## ETHICS STATEMENT

Animal studies were approved by the McGill University Animal Care Committee. All methods were performed in accordance with the relevant guidelines and regulations.

## Supporting information


Figure S1.
Figure S2.
Figure S3.
Figure S4.

Table S1.
