# Supplementary material for: Role of the Ste20‐like kinase SLK in podocyte adhesion
Source: Physiol Rep. 2024 Jan 1;12(1):e15897. doi: 10.14814/phy2.15897 (PMC10758337; doi:10.14814/phy2.15897)

Supplementary Figure Legends

Supplementary Figure S1. Potential SLK phosphorylation sites. A) The sites are taken from a database of peptides with serine and threonine sites that have a high probability of being phosphorylated by SLK (Johnson et al., 2023). The method for identification of each phosphopeptide in the present study and the results are indicated. MS, mass spectrometry; ND, not detected; phosphor, phosphorylation. B) Consensus sequence, adapted from (Johnson et al., 2023). Ω, aromatic; Φ, hydrophobic.

Supplementary Figure S2. Expression of vinculin, paxillin and talin in GECs. There are no significant differences between control and SLK KO GECs, and there are no significant effects of adriamycin treatment. Actin is the loading control. A representative immunoblot of 3 immunoblots is shown. Densitometry of each FAC protein is normalized to actin.

Supplementary Figure S3. Pearson correlation coefficients of talin and pFAK immunostaining in Fig. 4 (A), and vinculin and paxillin immunostaining in Fig. 3 (B). A) 32-38 cells per group in 2 experiments; B) 15 cells per group in 3 experiments. There are no significant differences among groups.

Supplementary Figure S4. Immunoblot of COS1 cells. COS1 cells were transfected with HA-SLK 1-373 or EGFP cDNAs (control). Lysates were immunoblotted with antibodies to HA or EGFP. A representative immunoblot of 2 immunoblots is shown.

A

Peptide		Method for Phosphopeptide Identification; Result
**Paxillin**		
S90	SSSPVYG**S**SAKTSSV	MS(mouse GECs; phosphor ND)
S250	SSPQRVT**S**TQQQTRI	Immunoblot (phosphor detected) & MS(peptide ND)
S119	GEEEHVY**S**FPNKQKS	MS(mouse GECs; phosphor ND)
S533	HYHERRG**S**LCSGCQK	MS(peptide ND)
**Talin-1**		
T144	KKEEITG**T**LRKDKTL	MS(COS1 & mouse GECs; phosphor ND)
T150	GTLRKDK**T**LLRDEKK	MS(peptide ND)
T354	VIQEWNL**T**NIKRWAA	MS(COS1 & mouse GECs; phosphor ND)
S405	IILKKKK**S**KDHFGLE	MS(peptide ND)
T430	SVSPKKS**T**VLQQQYN	MS(COS1 & mouse GECs; phosphor ND)
**Vinculin**		
S383	AMTNSKQ**S**IAKKIDA	MS(peptide ND)
T793	ASDELSK**T**ISPMVMD	MS(peptide ND)

B

SLK Consensus Sequence

X-X-K-Ω-R/K-**S/T**-Φ-R/K-R/K-X

Supplementary Figure 2

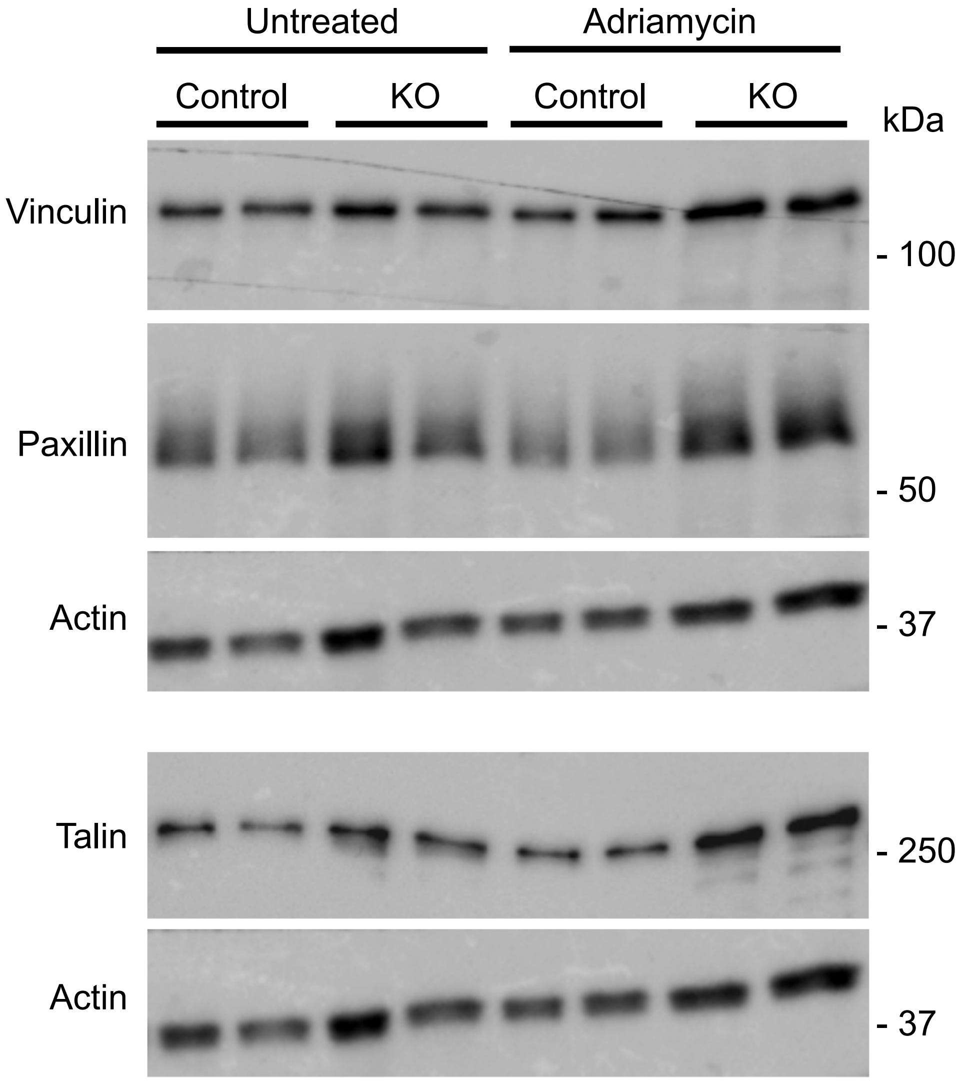

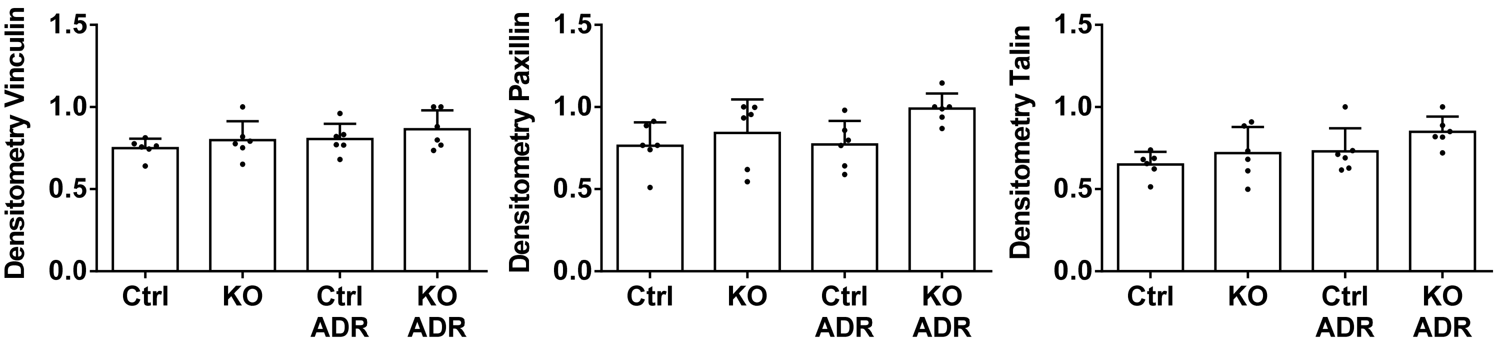

Supplementary Figure 3

A

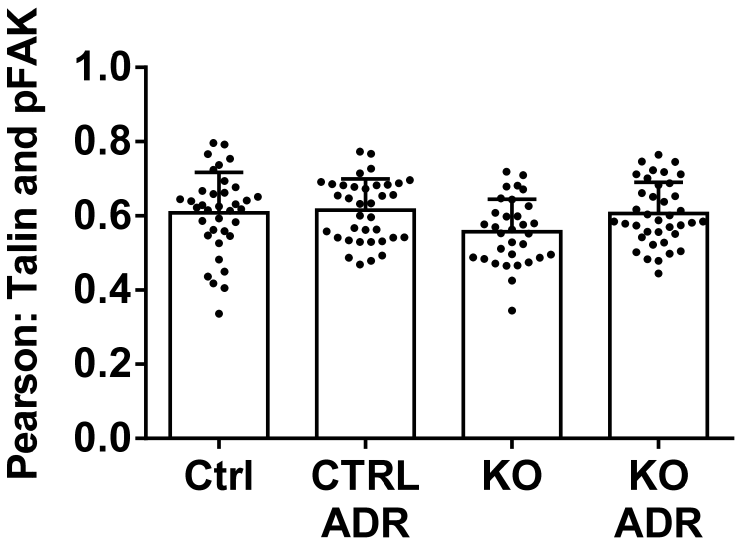

B

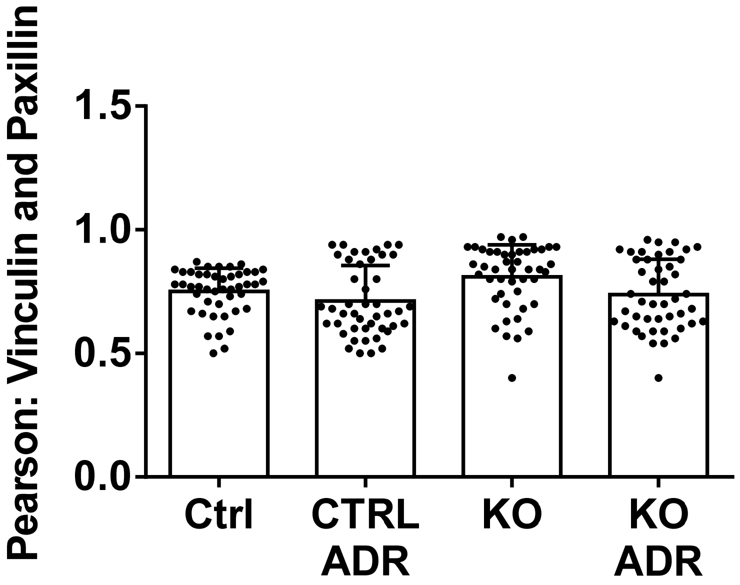

Supplementary Figure 4

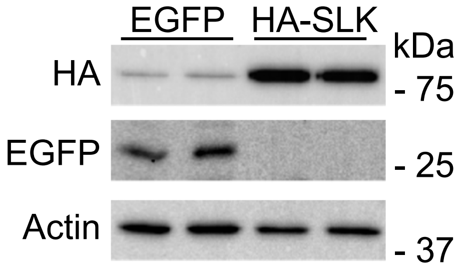

Supplement: Supplementary file 1 — Figure S1. Figure S2. Figure S3. Figure S4. [file PHY2-12-e15897-s001.pdf]
